# Unexpected excessive apixaban exposure: case report of a patient with polymorphisms of multiple apixaban elimination pathways

**DOI:** 10.1186/s40360-019-0331-9

**Published:** 2019-08-29

**Authors:** Andrea Huppertz, Caspar Grond-Ginsbach, Chris Dumschat, Kathrin I. Foerster, Jürgen Burhenne, Johanna Weiss, David Czock, Jan C. Purrucker, Timolaos Rizos, Walter E. Haefeli

**Affiliations:** 1 Department of Clinical Pharmacology and Pharmacoepidemiology, Heidelberg University Hospital, Im Neuenheimer Feld 410, 69120 Heidelberg, Germany; 2 Department of Neurology, Heidelberg University Hospital, Im Neuenheimer Feld 400, 69120 Heidelberg, Germany; 3 Department of Vascular and Endovascular Surgery, University Hospital Heidelberg, Im Neuenheimer Feld 110, 69120 Heidelberg, Germany

**Keywords:** Direct oral anticoagulants, Apixaban, Plasma concentration, Neurology

## Abstract

**Background:**

Apixaban effectively lowers the risk of ischemic stroke and systemic embolism in patients with non-valvular atrial fibrillation. Systemic exposure to a given apixaban dose depends on multiple clearance pathways. Though routine quantification of direct oral anticoagulants (DOACs) in neurological emergency situations has not been widely established, suspected associations of DOAC peak concentrations with bleeding events and DOAC trough concentrations with efficacy and safety suggest that such information might support clinical decision making.

**Case presentation:**

We describe the case of a 75 year-old woman with atrial fibrillation maintained on apixaban who was admitted due to suspected acute stroke. Clinical work-up did not confirm ischemic or hemorrhagic stroke but routine quantification of apixaban revealed an excessively high apixaban plasma concentration (~ 3 h after the last drug intake: 1100 ng/ml (expected range: 91–321 ng/ml); ~ 12 h after drug intake: 900 ng/ml (expected range: 41–230 ng/ml)) and a substantially prolonged elimination half-life (~ 31 h). The corresponding apixaban concentration-to-dose ratio was 9900 (ng/ml)/(mg/kg/d) and 8100 (ng/ml)/(mg/kg/d), respectively (expected range: 249–463 (ng/ml)/(mg/kg/d)). Renal function was only moderately impaired (creatinine 1.36 mg/dl (0.5–1.1 mg/dl), creatinine clearance 40 ml/min). Genotype analyses revealed that the patient was a CYP3A5*3/*3 non-expressor, a heterozygous carrier of the *ABCG2* c.421C/A alleles, and a homozygous carrier of *ABCB1* c.2677 T/T and *ABCB1* c.3435 T/T. In the absence of known drug interactions explaining apixaban clearance impairment, excessive apixaban concentrations were most probably caused by moderate renal impairment combined with multiple functional polymorphisms of apixaban clearance pathways.

**Conclusions:**

This case suggests that concurrent genetic polymorphisms can impair multiple apixaban elimination pathways and thus substantially increase its exposure.

## Background

Apixaban is a rapidly acting, direct concentration-dependent factor Xa (FXa) inhibitor of both free and prothrombinase-bound FXa [1–3]. It prevents thrombus formation [1–3], thereby considerably lowers the risk of ischemic stroke and systemic embolism in patients with non-valvular atrial fibrillation, and shows a good safety and tolerability profile [4–9]. Systemic apixaban exposure at a given dose depends on multiple clearance pathways involving renal elimination of unchanged drug (~ 23%) [10, 11] and oxidative metabolism by cytochrome P450 (CYP) isozymes, mainly CYP3A4 and polymorphic CYP3A5 (~ 20%) [10, 12] and excretion of unabsorbed apixaban in the gastrointestinal tract (approximately 34%) [10]. Linear relationships between apixaban concentrations and alteration of coagulation markers have been shown [13], at least for concentrations up to approximately 1000 ng/ml. The bleeding risk associated with very high concentrations remains unclear but is suspected to be considerably increased.

In our institution, quantification of direct oral anticoagulants (DOAC) in neurological emergency situations has been implemented in clinical routine, because their immediate action, the suspected association of peak concentrations with bleeding events [14], and the correlation of trough concentrations with efficacy and safety [15–17] suggest that such information can support clinical decision making.

## Case presentation

In November 2017, a 75-year-old Caucasian woman (body mass index 33 kg/m^2^) was admitted to our neurological emergency room with suspected acute stroke (weakness of her left leg, fall to the ground where she remained undetected for 12 h). She suffered from atrial fibrillation, arterial hypertension, type 2 diabetes mellitus, and hypothyroidism. Her medication comprised apixaban (2 × 5 mg/d; started in the outpatient setting by the general practitioner in 05/2016), ramipril 10 mg/d, candesartan 8 mg/d (CYP2C8 inhibitor), saxagliptin 5 mg/d, levothyroxine 100 μg/d alternating with 125 μg/d, and simvastatin 10 mg/d (CYP3A4 substrate). According to the prescribing list, the medicines taken ‘as needed’ were: amlodipine (when systolic blood pressure ≥ 160 mmHg), zopiclone 7.5 mg for insomnia, and pantoprazole 40 mg for ulcer prophylaxis.

**Fig. 1:**
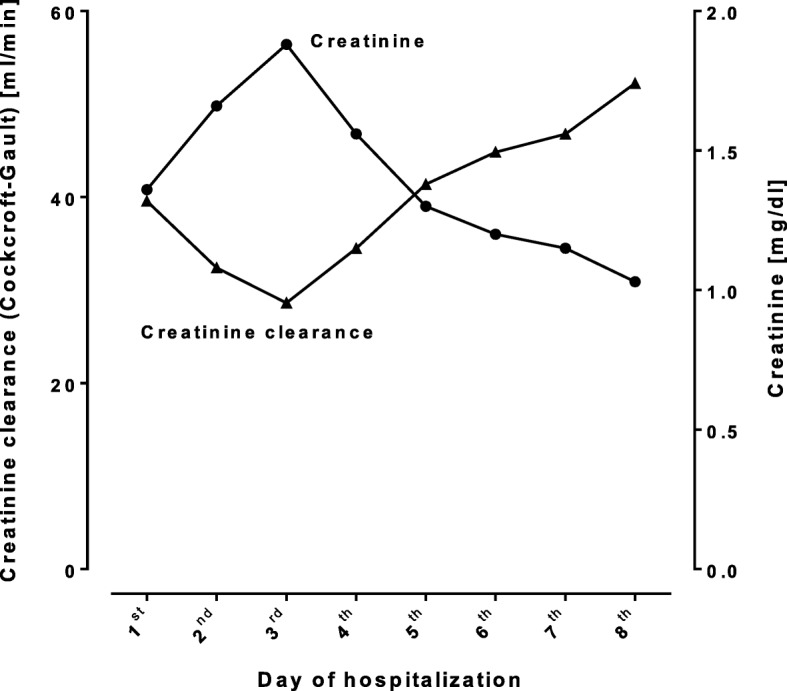
Time course of renal function during the hospital stay. The creatinine clearance was calculated using the Cockcroft-Gault formula with the adjusted body weight

**Fig. 2:**
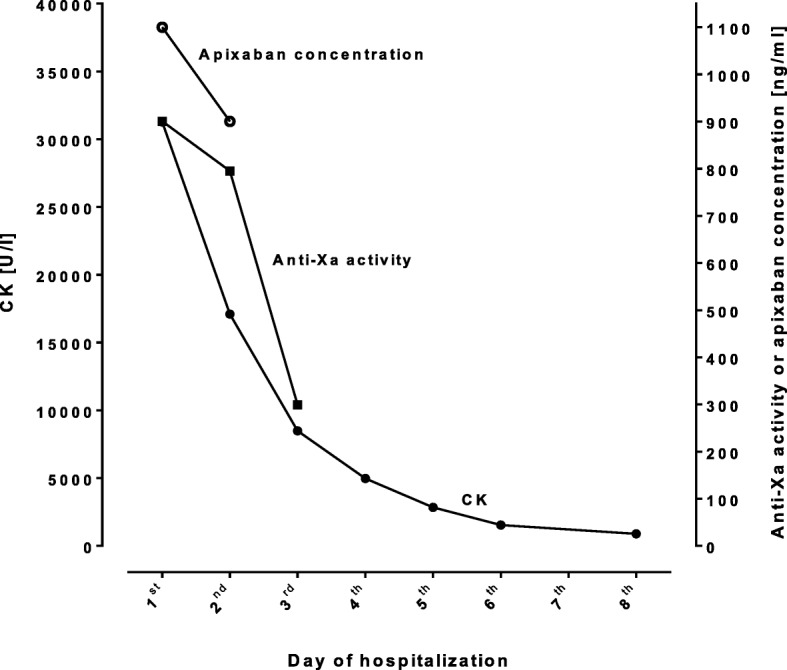
Time course of creatine kinase, calibrated anti-factor Xa activity, and apixaban concentrations measured by ultra-performance liquid chromatography-tandem mass spectrometry

The patient was transferred to the stroke unit. Ramipril, candesartan, and apixaban were stopped after admission and apixaban plasma concentrations were quantified using ultra-performance liquid chromatography-tandem mass spectrometry (lower limit of quantification, 1 ng/ml [18]). At admission, i.e. approximately 3 h after the last drug intake, the plasma concentration accounted for 1100 ng/ml (expected range: 91–321 ng/ml [19]). Approximately 12 h after drug intake, apixaban plasma concentration remained at high concentrations (900 ng/ml, expected range: 41–230 ng/ml [19]), revealing an elimination half-life of approximately 31 h. The corresponding apixaban concentration-to-dose (C/D) ratio was 9900 (ng/ml)/(mg/kg/d) 3 h after drug intake and 8100 (ng/ml)/(mg/kg/d) at trough (expected range: 249–463 (ng/ml)/(mg/kg/d) [20]). The FXa activity was measured by an anti-FXa assay calibrated for apixaban and resulted in somewhat lower but still very high apixaban concentrations (after 3 h and 12 h: 900 ng/ml and 795 ng/ml; Fig. 2). International normalized ratio values were elevated (INR: 1.58, 3 h after drug intake, and 1.45 after 12 h; upper limit of normal: < 1.2), whereas and as expected aPTT levels were within normal limits (33.3 and 30.8 s, reference range < 35 s) [13, 21]. Other laboratory results at admission revealed rhabdomyolysis (creatine kinase 31,269 U/l (normal < 170 U/l), ASAT 863 U/l (< 37 U/l)), moderate renal impairment (creatinine 1.36 mg/dl (0.5–1.1 mg/dl); estimated creatinine clearance 40 ml/min (Cockcroft-Gault formula with the adjusted body weight [22, 23]), and moderately elevated ALAT (186 U/l (< 35 U/l)) (Figs. 1 and 2).

After obtaining written informed consent, genotype analyses were performed to determine the CYP3A5 *3 (g6986A > G, rs776746) single nucleotide polymorphism (SNP), the ABCG2/BCRP SNP c.421C > A (rs2231142), and the *ABCB1*/P-gp SNPs G2677 T/A (rs2032582) and C3435T (rs1045642), which all have been associated with functional impairment of the corresponding gene product [20, 24–26]. Our patient was a CYP3A5*3/*3 non-expressor, a heterozygous carrier of the *ABCG2* c.421C/A, and a homozygous carrier of *ABCB1* c.2677 T/T and *ABCB1* c.3435 T/T.

During the hospital stay, ischemic and hemorrhagic stroke was ruled out by brain imaging. The diagnostic work-up revealed a left-sided sciatic nerve lesion, proven by electrophysiological examinations, being the cause of her leg weakness. Additional findings encompassed an asymptomatic stenosis of the right internal carotid artery (60–70%, NASCET criteria), urinary tract infection with *E. coli* (≥ 10^5^ colony-forming units/ml), and radiological signs of pneumonia accompanied by clinical infection signs and elevated laboratory inflammation markers (leukocytosis, C-reactive protein, and procalcitonin). Therefore, antibacterial therapy with metronidazole and ceftriaxone was started. After 10 days of hospitalization, the patient’s renal function improved (creatinine 1.03 mg/dl) (Fig. 2) and she was transferred to a rehabilitation center. In January 2018, apixaban was restarted at the rehabilitation clinic after renal function had normalized (dosage: 5 mg bid). Then, while taking apixaban, our patient suffered a duodenal/jejunal bleeding in May 2019. Transfusion of erythrocyte concentrates and interventional therapy was necessary and apixaban was paused. In June 2019, she was diagnosed with acute myeloid leukemia (AML) and, due to pancytopenia, no anticoagulation was restarted until today (end of June 2019). Unfortunately, apixaban concentrations or specific anti-Xa activity were not assessed.

## Discussion

**Fig. 3:**
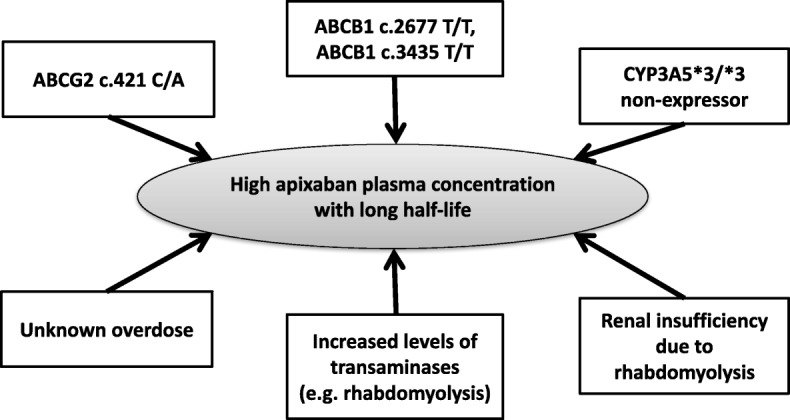
Summary of factors potentially leading to high apixaban plasma concentrations and a long half-life

Despite being regularly dosed with apixaban, our patient presented unexpectedly high plasma exposures and concurrent intense anticoagulation, which might be caused by a substantial impairment of apixaban elimination pathways. Various factors can have led to high apixaban plasma concentrations and the long half-life (Fig. 3), but no currently known drug interaction was present. Renal impairment prolongs elimination half-life [27, 28] but the effect of renal impairment on apixaban concentrations is rather mild and our patient’s renal function was only moderately impaired (clearance of 40 ml/min). According to Chang and co-workers [27], in patients with a creatinine clearance of 40 ml/min, the average increase in Cmax is 3.4% and in AUC 29%, i.e. substantially smaller than observed in our patient. Hence, the observed excessively high apixaban concentrations and the very long half-life in our patient are suggestive to be caused by additional factors. Apixaban has a high solubility and low passive permeability [19] and its intestinal absorption of apixaban is modulated by active efflux transporters. Apixaban is a substrate of BCRP/*ABCG2* [29] and also P-gp/*ABCB1* [24, 29], which modulate apixaban absorption, tissue distribution, and elimination at least in rats [30]. Several BCRP and P-gp polymorphisms leading to reduced transporter activities have been described in Caucasians [31], which might modulate relevant pharmacokinetic pathways of apixaban. In addition, apixaban’s major metabolites are mainly formed by the CYP3A enzyme family including the polymorphic CYP3A5 isozyme. In Japanese patients, BCRP and CYP3A5, but not P-gp polymorphisms were associated with higher apixaban trough concentrations [20, 26]. With every impaired elimination pathway, the percentage contribution to drug clearance of the remaining individual pathways increases, indicating that concurrent impairment of several minor pathways could ultimately cause major exposure changes as shown for rivaroxaban [32].

Our patient was a CYP3A5*3/*3 non-expressor, a heterozygous carrier of the *ABCG2* c.421C/A alleles, and a homozygous carrier of *ABCB1* c.2677 T/T and *ABCB1* c.3435 T/T. High apixaban exposures have already been reported in heterozygous and homozygous CYP3A5 non-expressors and in homozygous carriers of the deficient *ABCG2* c.421 A-allele, whereas heterozygous *ABCG2* c.421C/A carriers and *ABCB1* polymorphisms did not appear to influence apixaban pharmacokinetics [20, 26, 33]. However, thus far, apixaban pharmacokinetics of carriers of polymorphisms in all three pathways combined with moderate renal impairment has not been described. In sum, this genotype constellation could have led to a reduced clearance of apixaban. The clinical relevance of this finding remains unclear but such clearance changes might alter the risk/benefit relationship in affected patients because FXa plasma concentrations are directly linked to FXa inhibition [13] and efficacy and bleeding rates [17]. However, further studies to examine the genetic polymorphisms of apixaban metabolism and clinical outcome should be conducted particularly in patients with concurrent clearance impairment of other relevant drug elimination pathways such as the kidneys.

Our report has some limitations. First, the actually administered apixaban dose and time of drug intake are based on anamnestic information. Second, renal function was estimated using the Cockcroft-Gault equation, which overestimates renal function in patients with acute kidney injury. Thus, actual renal function was probably lower than the estimated value. However, the effects of renal impairment on apixaban pharmacokinetics are limited [27]. Finally, we assume that transient renal impairment was the only effect of rhabdomyolysis on apixaban pharmacokinetics; whether rhabdomyolysis can affect other apixaban clearance pathways is currently unknown.

## Conclusion

We cared for a patient on oral anticoagulation with regular apixaban doses who had excessively high apixaban exposures and a substantially prolonged elimination half-life. The impaired clearance was most probably the result of moderate renal impairment combined with multiple functional polymorphisms of apixaban clearance pathways that are all partially involved in apixaban disposition and in sum may have caused substantial drug accumulation. Therefore, genotype analyses should be considered in patients with otherwise unexpected high plasma concentrations of apixaban.

## Data Availability

All the data supporting our findings are contained within the manuscript.

## Abbrevations

ALAT: Alanine aminotransferase
AML: Acute myeloid leukemia
aPTT: Activated partial thromboplastin time
ASAT: Aspartate aminotransferase
BCRP: Breast cancer resistance protein
CYP: Cytochrome P450
DOAC: Direct oral anticoagulants
FXa: Factor Xa
INR: International normalized ratio
NASCET: North American Symptomatic Carotid Endarterectomy Trial
P-gp: P-glycoprotein
SNP: Single nucleotide polymorphism
